# Influence of Curing Agent Amount on Properties of Dynamic Vulcanized Phenyl Silicone Rubber-SEBS-SBS System

**DOI:** 10.3390/polym14245443

**Published:** 2022-12-12

**Authors:** Chunxu Zhao, Bobing He, Xian Chen

**Affiliations:** College of Chemistry, Sichuan University, Chengdu 610065, China

**Keywords:** thermoplastic vulcanized rubber, methyl vinyl phenyl silicone rubber, backscattered electrons, dynamic vulcanization, scanning electron microscope

## Abstract

In this paper, we prepared a new type of thermoplastic vulcanizate (TPV) by melt blending methyl vinyl phenyl silicone rubber (PSR), styrene butylene copolymer (SBS), and hydrogenated SBS (SEBS) and then dynamically vulcanizing it. At the same time, we studied the influence of the content of the vulcanizing agent on the properties. The corresponding backscattered electron images were obtained by a scanning electron microscope (SEM) test of each group of samples, as well as the distribution of the PSR phase and the SEBS-SBS phase, and the vulcanization process of the samples with a vulcanizing agent content of 1 phr were characterized. According to the imaging principle of the backscattered electron signal, we found that the atomic number contrast can be clearly reflected in the backscattered image. From the obtained images, we found that PSR is a dispersed phase, while SEBS and SBS are continuous phases, that is, they had a “Sea-Island” structure. In the first 30 s of the vulcanization reaction, the “Sea-Island” structure is formed, and then the vulcanization reaction rate gradually slows down. We then printed the images and analyzed them using a colorimeter and found that it was feasible to quantitatively characterize the size of the compatible layer between the continuous and dispersed phases. According to the quantitative characterization results, we found that the silane coupling agent KH-172 can increase the thickness of the compatible layer by nearly 35%. In addition, we also tested the mechanical properties and low-temperature elastic properties of the material. Finally, we found that when the content of the vulcanizing agent was 1 phr, the elastic properties and tensile properties were the best, and when the content of the vulcanizing agent was more than 1 phr, the tensile and elastic properties of the material decreased significantly. At the same time, we also found that the addition of the silane coupling agent KH-172 can also significantly improve the tensile properties and elastic properties of TPV, which we believe is related to the increase in the thickness of the compatible layer. The test results of dynamic mechanics show that PSR has good compatibility with SEBS-SBS. When the vulcanizing agent content is less than or equal to 1 phr, the material exhibits good low-temperature resistance. In addition, through the test of the melt index of each group, it was also found that the addition of the vulcanizing agent will affect the fluidity of the melt to a certain extent. When the content of the vulcanizing agent is greater than 1 phr, the melt fluidity decreases more obviously.

## 1. Introduction

**Figure 1:**
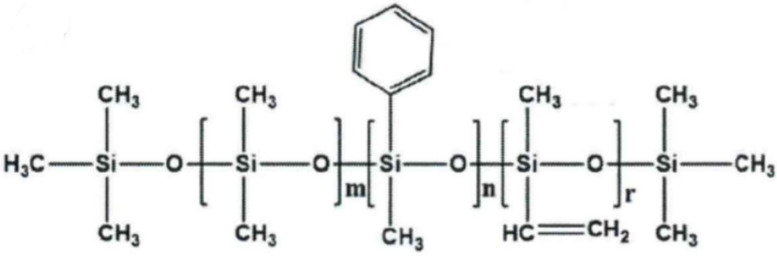
The structural formula of methyl vinyl phenyl silicone rubber.

Silicone rubber is a polyorganosiloxane whose main chain is composed of silicon and oxygen. However, silicone rubber is divided into different types, according to the branched chain [1]. Figure 1 shows the structural formula of methyl vinyl phenyl silicone rubber (PSR). According to the ratio of the number of moles of phenyl-containing chain units to the number of moles of all chain units, we often designate phenyl silicone rubber with a mole fraction less than 10% as low phenyl silicone rubber [2]. It has attracted much attention because of its good elasticity and low-temperature resistance [3,4]. However, its application conditions are limited due to its poor mechanical strength [5]. Blending silicone rubber with other polymers is a common modification method [6,7,8]; thus, we plan to prepare a modified thermoplastic elastomer (TPE) by blending silicone rubber with SBS and SEBS through dynamic vulcanization. Generally, TPE prepared by dynamic vulcanization is called thermoplastic vulcanized rubber (TPV) [9]. The first developed TPV is made of ethylene propylene diene monomer (EPDM) and polypropylene (PP), and it was the first to start the industrial production of this TPV by Monsanto [10,11,12]. SBS is a block copolymer made from the polymerization of styrene and butadiene. It is often used as a modifier of asphalt and has good tensile strength, low-temperature performance, and processability [13,14]. SEBS is the product of SBS hydrogenation; hence, in addition to the advantages of SBS, SEBS also has good aging resistance, ozone resistance, and other properties [15,16,17]. In this paper, the modified TPV we prepared is a new material with the advantages of these three polymers. In the preparation process of this material, process conditions, such as temperature and rotor speed, and the content of the different substances in the formulation will affect the performance of the TPV [18,19]. However, since we prepare materials by dynamic vulcanization, and vulcanization is an indispensable step to impart performance to rubber [20], it is very important to study the effect of the vulcanizing agent content on the TPV performance and find the appropriate vulcanizing agent content.

As mentioned above, our method for preparing this novel modified TPV is dynamic vulcanization. This is a process in which rubber and thermoplastics are melted and blended in a high-temperature, high-shear mixer, and the vulcanized rubber is broken up by high-shear force to disperse it well in the thermoplastic component [21,22]. Therefore, it is necessary to observe the distribution among the phases and find the correlation with the macroscopic properties. Usually, for the phase distribution study of polymer blends, researchers often use the etching method. This is a method of operation that dissolves a phase in a polymer blend and takes a secondary electron signal map of the undissolved phase [23]. In this way, we see to some extent the distribution of the polymer and the interaction between the phases. However, this method has the disadvantages that it is difficult to find a good solvent for a certain phase and it is difficult to determine the complete dissolution [24]. Therefore, to better characterize the distribution of different phases, in this paper, we use the image generated by the backscattered electron signal of the scanning electron microscope to characterize the phase distribution, and we also creatively characterize the thickness of the compatibility layer between the two phases quantitatively. Since the backscattered electron signal is related to the atomic number [25,26,27], the characterization method of the backscattered electron signal map is often used to observe the phase change of the alloy [28,29] and the phase distribution of the cement section [30,31]. When this characterization method is applied to the observation of polymer blends, for polymer blend systems with no difference in atomic number, researchers often dye the samples to make the characterization results of the backscattered electron signal clear [32,33]. There are also reports on the characterization of the three-dimensional structure of polymer blends using this method [34]. In this case, however, there are differences in atomic numbers between the components in the blend, thus we can obtain good results without dyeing. In addition, backscattered electron images can also overcome the shortcomings of secondary electron image characterization methods, such as difficulty finding a good solvent for the etched phase and the need to destroy the sample for testing [35,36].

## 2. Experimental

### 2.1. Materials

Methyl vinyl phenyl silicone rubber (LE-1150, Ron Silicon Material Co., Ltd., Nanjing, China), SEBS (YH-602T, Baling Petrochemical Branch of Sinopec Group, Yueyang, China), SBS (D1155JOP, Kraton, Belpre, OH, USA), white oil (250N, Wanghai Petrochemical Co., Ltd., Taizhou, China), silane coupling agent KH-172 (vinyltris(2-methoxyethoxy)silane, Yuanjin New Material Co., Ltd., Qufu, China), platinum catalyst (Daxi Chemical Raw Materials Co., Ltd., Guangzhou, China), and hydrogen-containing silicone oil (hydrogen content 1.6%, Xinglongda New Materials Co., Ltd., Jinan, China).

### 2.2. Sample Preparation

According to the formulations shown in Table 1, the samples differed only in the hydrogen-containing silicone oil content. Before adding the sample to the torque rheometer, we need to use an overhead electric mixer (Kexing Instrument Co., Ltd., Shanghai, China) to pre-mix the SBS, SEBS, and white oil (the total volume of the mixture is about 190 mL) at a speed of 100 rpm, and then let stand for 30 min, in order for the white oil to be more fully absorbed by the SBS and SEBS. Subsequently, we added the previous mixture into the torque rheometer and added phenyl silicone rubber after the torque value was stable. The temperature in the torque rheometer chamber was 170 °C, and the rotor speed was 70 rpm. After the torque value reaches equilibrium again, we sequentially add a silane coupling agent, hydrogen-containing silicone oil, and platinum catalyst to let the vulcanization reaction start until the torque value reaches equilibrium, which means that the vulcanization reaction is basically over. After the vulcanization reaction, we take out the mixture from the mixing chamber. Then we put the mixture into a thermocompression molding machine for processing, and after processing, a sheet with a size of 146 × 144 × 2 mm^3^ was obtained for further experiments.

### 2.3. Scanning Electron Microscope Test

A total of 5 rectangular specimens (dimensions of 10 × 2 × 2 mm^3^) were cut out from the 5 sheets prepared before, and then we clamped them with tweezers and placed them in liquid nitrogen to freeze for 3 min. We then took it out, broke it immediately, and sprayed gold on the cross-section after the sample was broken. The gold-sprayed samples could be imaged using a scanning electron microscope (Thermo Fisher Scientific, Helios G4 UC, Waltham, MA, USA). When observing the vulcanization process, it is not necessary to press the molten blend into tablets. To ensure the accurate vulcanization time of the characterization, we immediately took out the blend from the torque rheometer cavity and put it into liquid nitrogen for cooling. After quenching and spraying gold, the corresponding images were observed.

### 2.4. Fourier Transform Infrared Spectroscopy Test

We took a sample of size 10 × 8 × 2 mm^3^ from the prepared sheet and processed the sample using the tableting method. After the sample was processed, it was tested by Fourier transform infrared spectroscopy (Bruker Scientific Technology Co., Ltd., Beijing, China). The relevant parameters are: the number of scans is 16, and the detection mode is ATR with a resolution of 4 cm^−1^.

### 2.5. Compatibility Layer Quantitative Characterization Test

**Figure 2:**
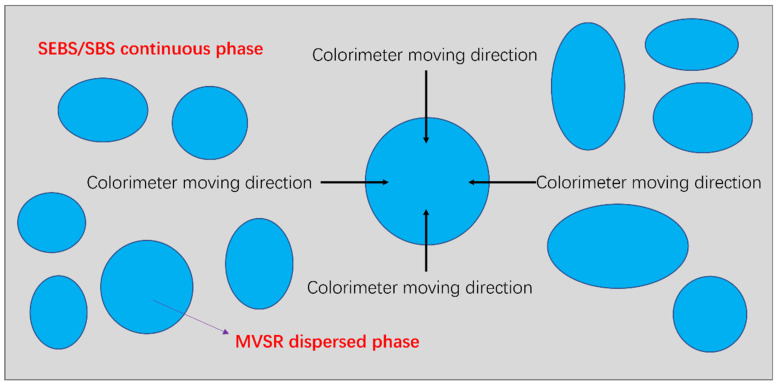
The test method for the colorimeter to test the size of the compatibility layer in TPV.

The 2000-times backscattered electron image (image pixel: 3840 × 2160 dpi) obtained by the scanning electron microscope test was printed on photo paper by a high-definition printer. The pixel of the printer is 1200 × 1200 dpi, and the size of the photo paper is 420 mm × 297 mm. We first tested the color space coordinates of the bright and dark parts of the image. Then we put the light port of the colorimeter in the dark place of the backscattered image; moved the colorimeter in the direction shown in Figure 2, moving 1 mm each time; and recorded the color space coordinate values here for further analysis of the data. Generally, the faster the color space coordinate value changes, the smaller the thickness of the compatible layer and the weaker the interaction between the two phases. It should be noted that to reduce the test error, at least four directions should be tested for each disperse phase tested.

### 2.6. Mechanical Property Test

We used a dumbbell-shaped cutter to cut the original sample. After cutting, we obtained 5 dumbbell-shaped samples with a gauge length of 20 mm, a width of 4 mm, and a thickness of 2 mm. The reference standard is GB/T 528-2009. The mechanical properties of each dumbbell-shaped specimen were then tested using a tensile testing machine (AGS-J, Shimadzu Corporation, Kyoto, Japan), where the tensile rate was 200 mm min^−1^. To test the tensile set of the experiment, we placed the broken specimens for about 5 min, after which the gauge length of the specimens was measured. The arithmetic means of the values of each group of mechanical property tests were taken as the result.

### 2.7. Dynamic Mechanical Properties Test

We cut out rectangular samples with a length of 35 mm, a width of 8 mm, and a thickness of 2 mm from the 5 sets of samples prepared in Section 2.2. The storage modulus and tangent value of the loss angle of the sample at different temperatures were tested by a dynamic thermomechanical analyzer (Q850, Walters Technology Co., Ltd., Shanghai, China), in which the test temperature range was −100 °C to 0 °C, and the heating rate was 5 °C min^−1^. The type of clamp used is the tensile clamp. 

### 2.8. Low-Temperature Hardness Test

We put the 5 groups of samples prepared in Section 2.2 into an ultra-low temperature refrigerator, adjusted the temperature of the refrigerator to −60 °C, and then froze for 72 h. After that, the hardness test was carried out, and the hardness tester was placed in the refrigerator for 30 min before the test. Each group of samples tested 10 groups of data to take the arithmetic mean. In order to prevent a temperature rise in the refrigerator caused by continuous testing, which could affect the test results, we put the hardness tester into the refrigerator after testing 3 sets of data, closed the refrigerator door, and let it stand for 10 min before subsequent testing.

### 2.9. Melt Index Test

We took the melt blend prepared according to Table 1 out of the torque rheometer and put it into a melt flow rate tester (XRN-400C, Jinjian Testing Instrument Co., Ltd., Chengde, China) to preheat for 10 min. After, we started the melt index test. The test temperature was 190 °C and the load weight was 5 kg.

## 3. Results and Discussions

### 3.1. Torque Variation Diagram and Infrared Spectrum Analysis of Modified TPV Made of PSR and SEBS-SBS with Different Vulcanizing Agent Contents

**Figure 3:**
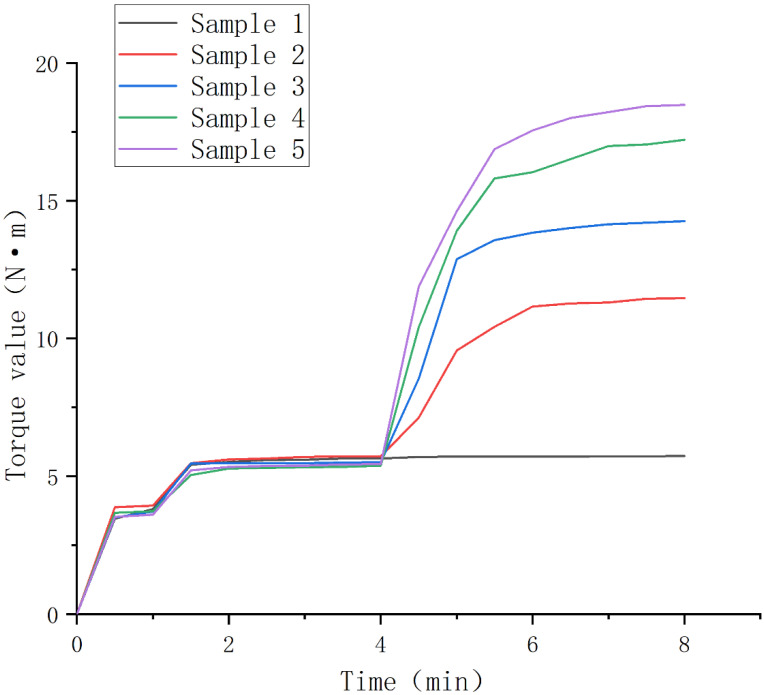
Torque value change diagram of samples 1–5 during vulcanization.

The torque variation diagram of samples 1–5 is shown in Figure 3. Sample 1 is a simple blended TPV control group without a vulcanizing agent. When no vulcanizing agent is added, the torque value is balanced around 5.5 Nm. After adding the vulcanizing agent, it is not difficult to see that with increasing amounts of vulcanizing agent added, both the torque value and the equilibrium torque value increase after vulcanization. Because the vulcanization reaction is a cross-linking reaction that occurs inside the polymer, which is microscopically manifested as the formation of a three-dimensional network structure, the torque value increasing speed can be regarded as a characterization of the intensity of the cross-linking reaction inside the torque rheometer. From this, we see that the experimental group with the addition of 2 phr vulcanizing agents has the most severe cross-linking reaction, but as the increase of vulcanizing agent is equal between each group, we also find that the increased rate of the torque graph rise rate is not uniform. When the content of the vulcanizing agent increased from 0 phr to 1 phr, the increase of the cross-linking reaction rate was the most obvious, while the increase of the cross-linking reaction rate decreased when the content of the vulcanizing agent increased from 1 phr to 2 phr. In addition, we also find that with the increase of the vulcanizing agent content, the time for the vulcanization system to reach torque equilibrium will decrease, that is, the time of the vulcanization reaction decreases.

**Figure 4:**
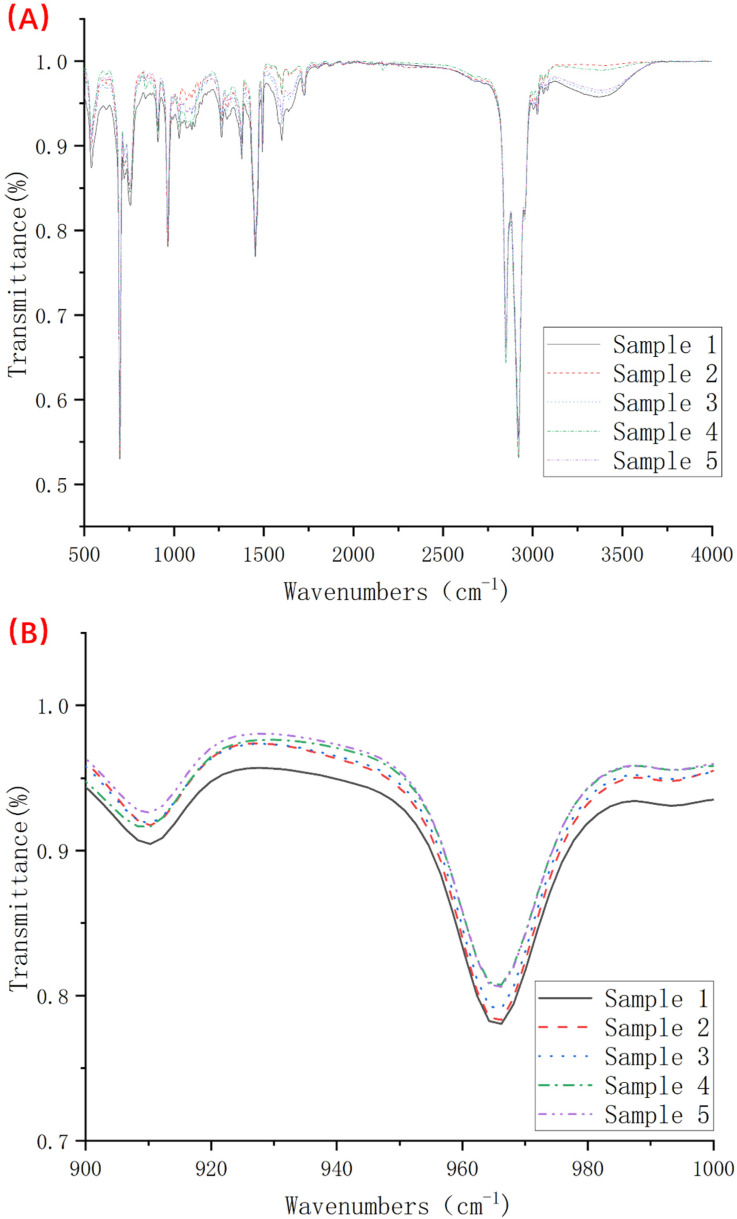
Infrared spectra of samples 1–5 (where (**A**) is the complete spectrum and (**B**) is the detailed image of the wave number 900–1000 cm^−1^).

In addition, we carried out infrared spectroscopy tests on samples 1–5, and the test results are shown in Figure 4. From Table 2, we know that the wavenumbers of 910 cm^−1^ and 966 cm^−1^ correspond to the characteristic absorption peaks of the 1,2 chain unit and the characteristic absorption peak of the anti-1,4 chain unit, respectively. As the essence of hydrosilylation was a hydrogenation reaction of double bonds, we found that the transmittance of each group of samples at wave numbers 910 cm^−1^ and 966 cm^−1^ as in Figure 4B was significantly different. When the content of the vulcanizing agent is 0.5–1.5 phr, the degree of hydrogenation of the 1,2 chain segment has almost no change, and only after further addition of the vulcanizing agent can the degree of hydrogenation at the 1,2 chain segment be increased. The situation at 966 cm^−1^ is completely different from that at 910 cm^−1^. When the vulcanizing agent content increases from 0.5 phr to 1.5 phr, the degree of hydrogenation of the anti-1,4 chain units increases to varying degrees. Interestingly, after further increasing the amount of the vulcanizing agent, the degree of hydrogenation does not change, but hydrogenation occurs at the 1,2 chain link. In general, increasing the amount of vulcanizing agent can make the hydrogenation degree of this TPV system continue to increase, but there is a difference in the hydrogenation position. 

### 3.2. The Backscattered Electron Images of TPV Made of PSR and SEBS-SBS with Different Vulcanizing Agent Contents and Analysis of the Characterization Results of the Vulcanization Process

Scanning electron microscopy is a characterization method that obtains information about the surface of a sample by analyzing the electronic signal excited by the interaction of incident electrons with the sample. Etching, which is commonly used for the microscopic characterization of polymeric materials, uses the secondary electron signal, while the scanning electron microscope images taken in this paper use the backscattered electron signal. As the intensity of the backscattered electron signal is related to the atomic number, when the atomic number is less than 20, the relationship between the signal intensity and the atomic number is almost linear. Therefore, the silicon-dominated PSR phase and the carbon-dominated SEBS-SBS phase in TPV can be clearly distinguished in the backscattered images [37].

**Figure 5:**
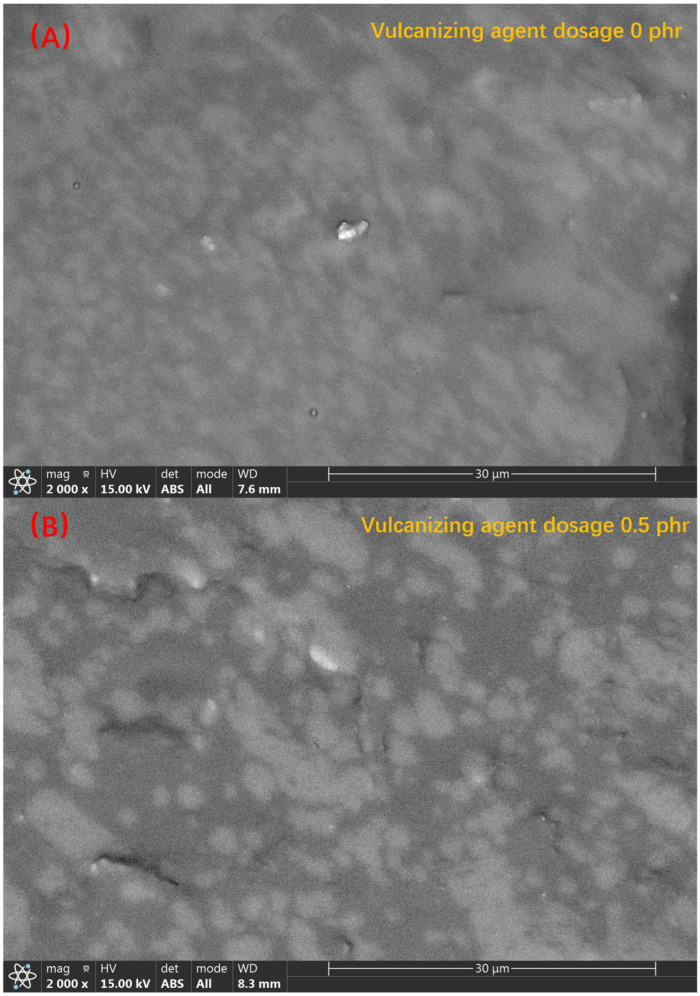

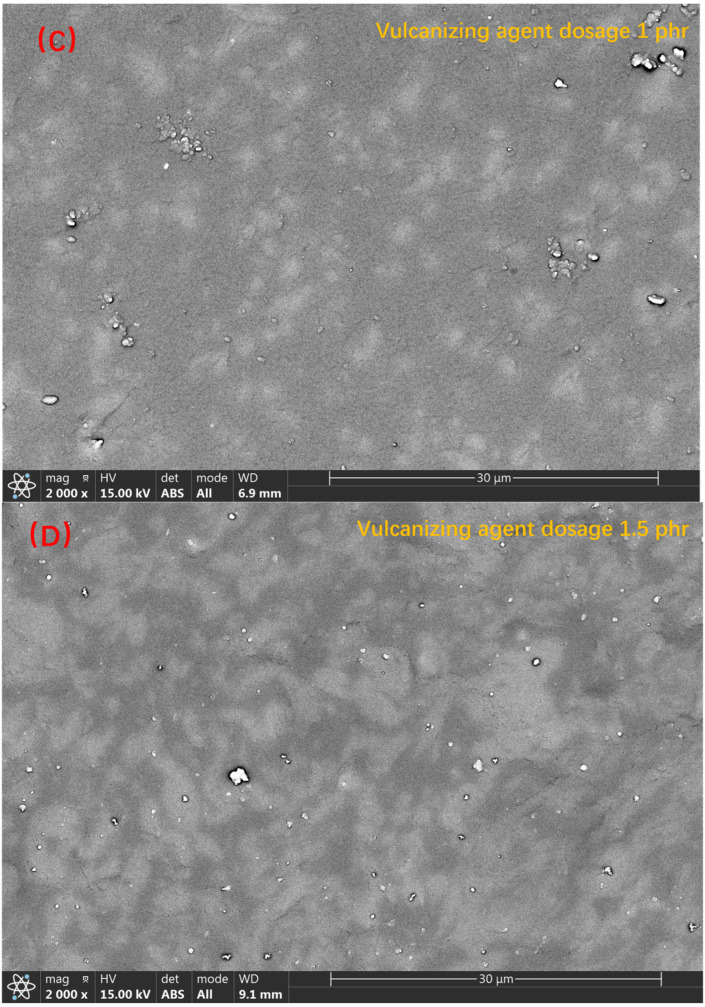

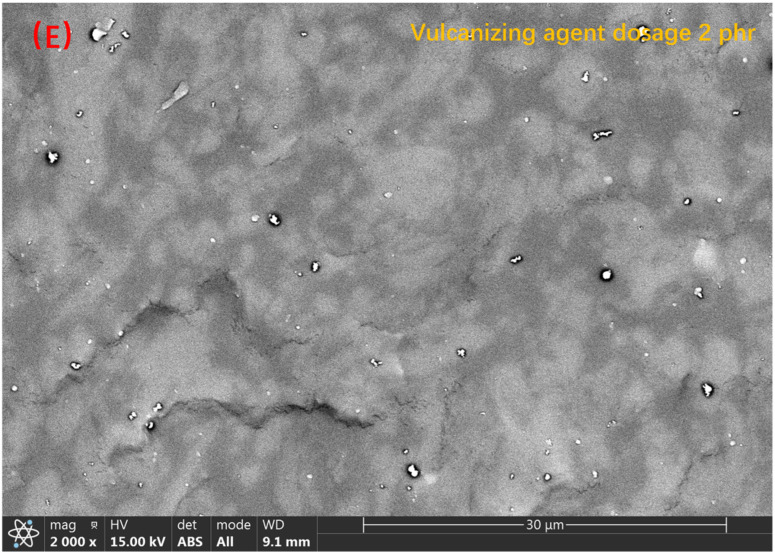
Backscattered electron images of samples 1–5 ((**A**–**E**) correspond to samples 1–5, respectively).

Figure 5 shows the 2000-times backscattered electron images of samples 1–5. According to the above-mentioned backscattered electron imaging principle, we clearly find that the PSR phase and the SEBS-SBS phase have a clear degree of distinction. From Figure 5A, we found that the PSR phase and the SEBS-SBS phase in the simple blend TPV without vulcanizing agent were in a co-continuous state, and it was impossible to clearly distinguish which was a continuous phase and which was a dispersed phase, which was particularly obvious in the right half of the image. When the amount of vulcanizing agent added was increased to 0.5 phr, a “Sea-Island” structure began to appear between the PSR phase and the SEBS-SBS phase, in which the PSR phase was dispersed as a dispersed phase between the continuous phases composed of the SEBS-SBS phase. However, interestingly, from the middle and bottom right of Figure 5B, we see that the dispersion of the PSR phase in the SEBS-SBS phase is not very uniform. When the amount of vulcanizing agent is increased to 1 phr, we find from Figure 5C that the dispersion uniformity of the PSR phase in the SEBS-SBS phase is significantly better than that of sample 2, and the “Sea-Island” structure is also clearer. However, when we continued to increase the content of the vulcanizing agent, we were surprised to find that the dispersion of the dispersed phase became significantly worse. Through Figure 5D,E, we see that a large number of PSR phases agglomerate together, which makes the co-continuous structure between the PSR phase and the SEBS-SBS phase more inclined. This shows that the vulcanizing agent has a certain promoting effect on the formation of the “Sea-Island” structure in TPV, but this effect does not increase with the increase of the vulcanizing agent content. In this experiment, the vulcanizing agent content of 1 phr is the most suitable.

**Figure 6:**
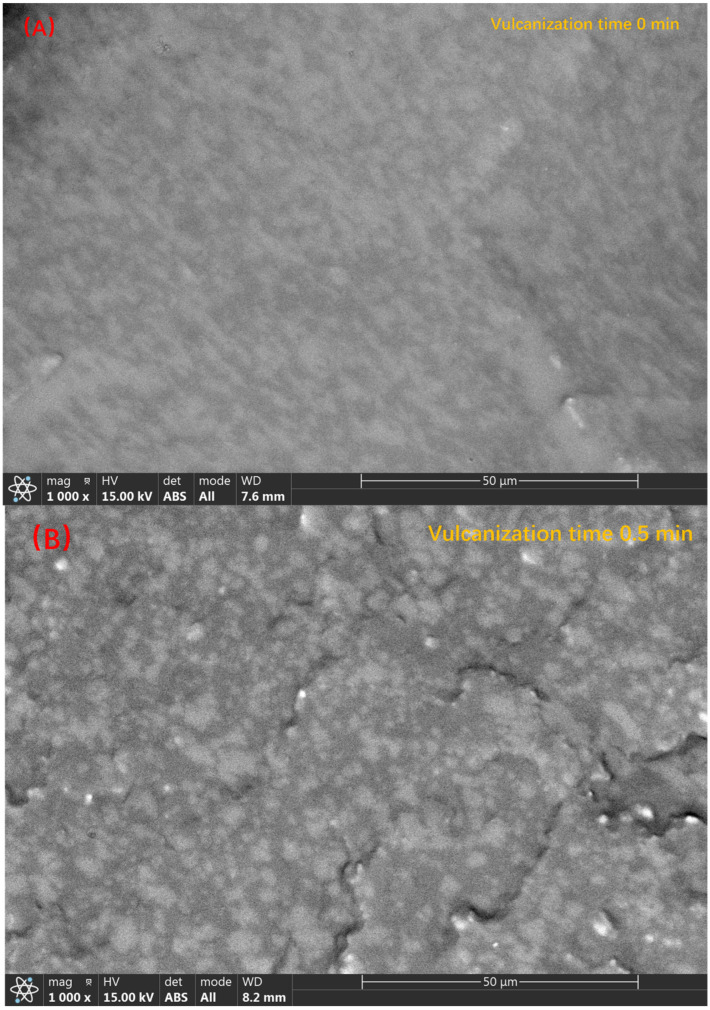

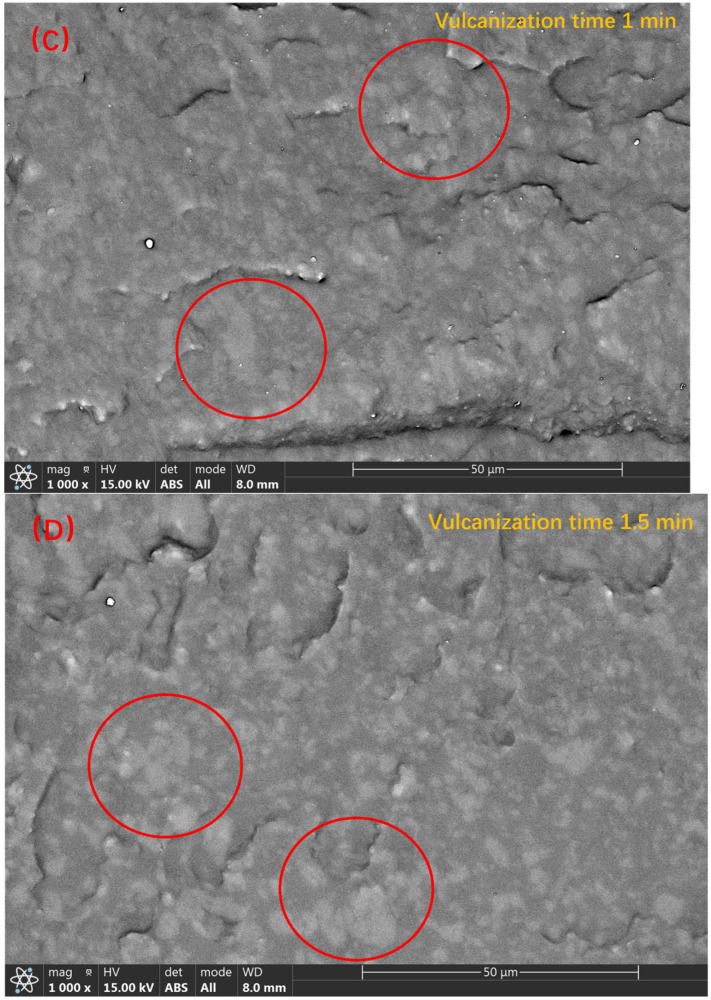

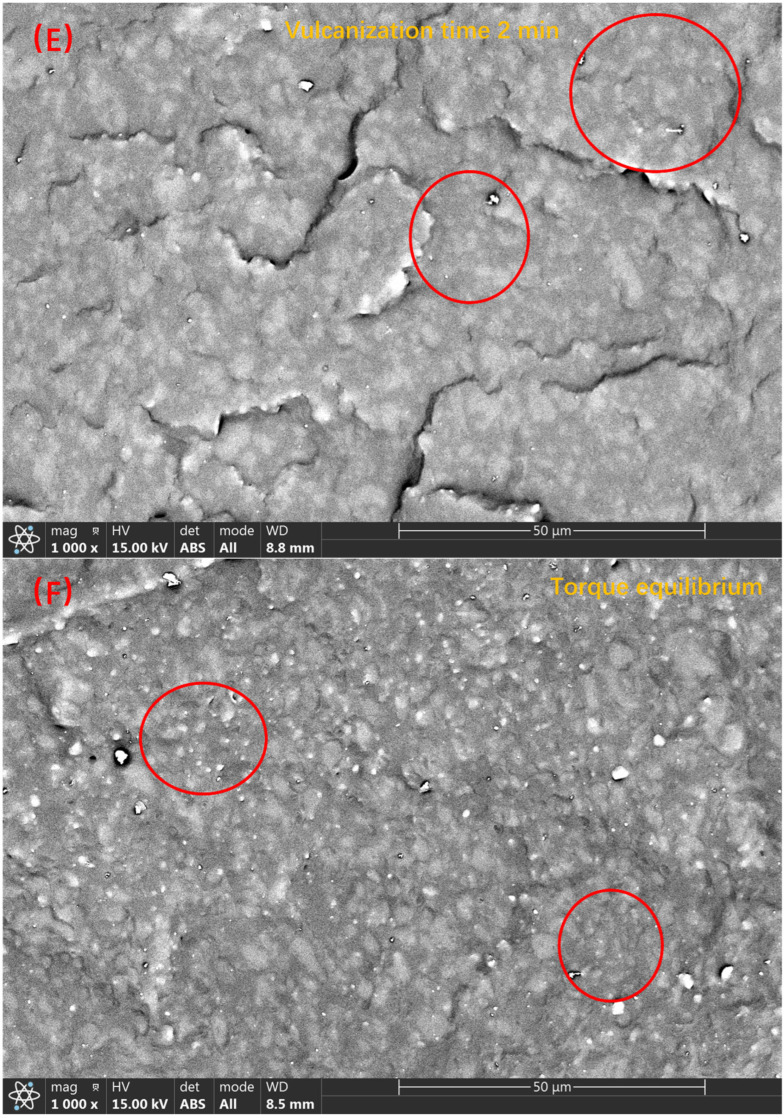
The backscattering image of sample 3 during the vulcanization process ((**A**–**E**) correspond to the vulcanization times of 0 min, 0.5 min, 1 min, 1.5 min, and 2 min, respectively, and (**F**) is the backscattered image after torque equilibrium. The change in the particle size of PSR as the vulcanization process proceeds is marked by the circles in the figure).

Next, we characterize the formation process of the “Sea-Island” structure. As shown in Figure 6, the 1000-times backscattered images of sample 3 taken at different curing times, we see the formation process of the “Sea-Island” structure. Initially, when no vulcanizing agent was added, as shown in Figure 6A, the PSR phase and the SEBS-SBS phase exhibited a co-continuous structure, and we could not distinguish the continuous phase from the dispersed phase. When the vulcanization reaction occurs for 0.5 min, according to the change of torque value in Figure 3, we see that the torque increases rapidly, which means that the initial reaction of the vulcanization reaction is rapid. Combined with Figure 6B, we found that the change at the microscopic level corresponding to the torque value at this time is the formation of a “Sea-Island” structure, in which the PSR phase is the dispersed phase and the SEBS-SBS phase is the continuous phase. After, with the progress of the vulcanization process, when the vulcanization reaction occurred for 1–1.5 min, the smaller diameter PSR phase was more evenly distributed in the SEBS-SBS phase, but the larger diameter PSR phase marked in Figure 6C,D still existed. When the vulcanization reaction continues, we see that the diameter of the PSR phase decreases further through the marks in Figure 6D,E, and the PSR phase with a larger diameter almost disappears in the observation field. According to Figure 3, the torque value change tends to be stable at this time, which means that the vulcanization reaction tends to end. The reason for the smaller diameter of the PSR phase and the more uniform dispersion is that the rotation of the rotor applies an external shear force to the blend such that the dispersed phase is more uniformly dispersed in the continuous phase.

### 3.3. Analysis of the Results of the Size Chart of the Compatibility Layer and Mechanical Property Test Results

We often use color space coordinates to describe different colors, and each color has a one-to-one correspondence with its color space coordinates. This coordinate consists of the three variables *L*, *A*, and *B*, representing black and white, red-green, and yellow-blue, respectively. In this paper, the backscattered electron image we obtained is black and white; hence, we only use the *L* value to describe the color of different positions in the image. To quantitatively characterize the thickness of the compatible layer, we tested the *L* value of the continuous phase and the dispersed phase and found that the *L* value of the continuous phase was 70–72, and the *L* value of the dispersed phase was 47–50. Therefore, we take the distance with an *L* value of 50–70 as the dimension of the compatibility layer.

We printed 2000-times BSE images on A3 size (420 mm × 297 mm) photo paper with a high-definition printer and moved the colorimeter as shown in Figure 1. Through the scale conversion of backscattered images, we calculated that the field of view of each backscattered image is 63.91 × 41.55 μm^2^ and that the size corresponding to each 1 mm movement of the colorimeter is 0.1522 μm. In each direction, every time we moved the colorimeter by 1 mm, we recorded the *L* value once and considered the arithmetic average of the thickness of the compatible layer obtained from each direction as the final experimental result.

**Figure 7:**
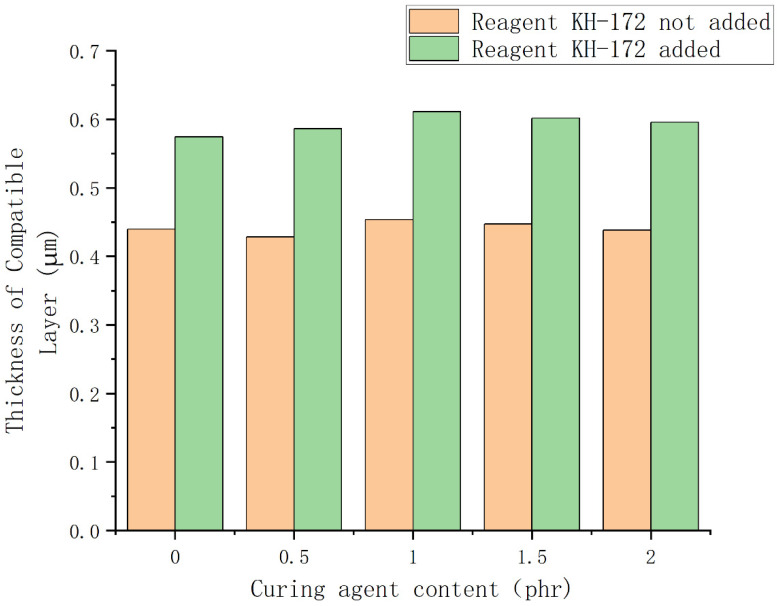
Thickness histogram for quantitative characterization of the compatible layer.

**Figure 8:**
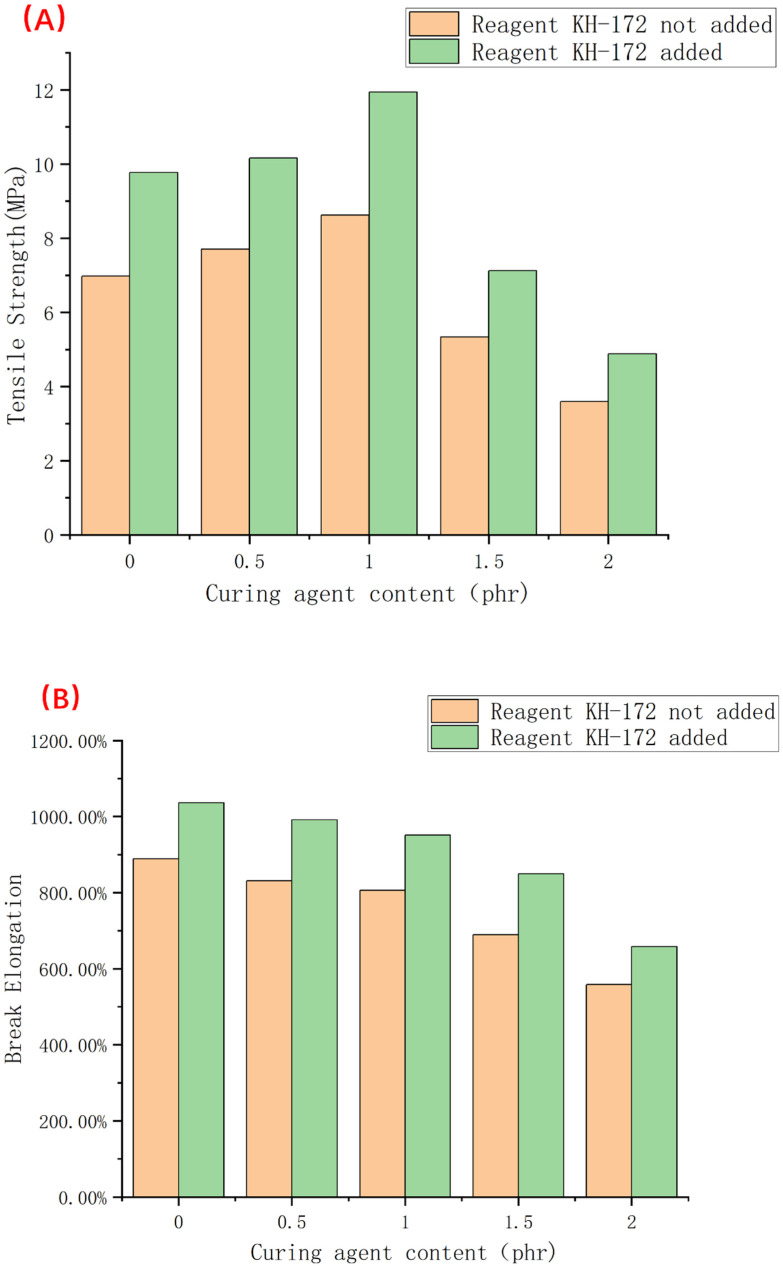
Histogram of tensile strength and elongation at the break of samples 1–5 ((**A**) is the change in tensile strength of each group of samples before and after adding KH-172, and (**B**) is the change of elongation at break of each group of samples before and after adding KH-172.).

**Figure 9:**
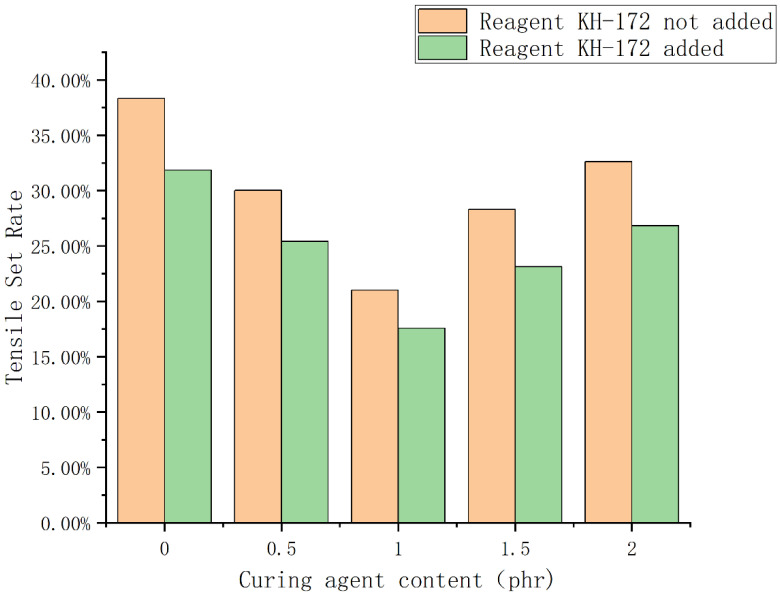
Histogram of tensile permanent deformation of samples 1–5.

According to the principle described above, we tested the thickness of the compatible layer on the backscattered electron images of samples 1–5. At the same time, to test the effect of the silane coupling agent KH-172 on the formula, backscattered electron images were also taken for the control group without KH-172, and the thickness of the compatibility layer was tested. The results are shown in Figure 7. From Figure 7, we find that the use of the vulcanizing agent has little effect on the thickness of the compatibility layer between the two phases. At the same time, we find that KH-172 can effectively increase the thickness of the compatibility layer between the two phases. After calculation, the thickness of each group of compatible layers can be increased by nearly 35% after adding KH-172. In addition, we also tested the mechanical properties of the samples with and without KH-172 added, and the test results are shown in Figure 8 and Figure 9. From Figure 8, we see that the addition of KH-172 can effectively increase the tensile strength and elongation at the break of the material. It should be noted that the tensile permanent deformation is an index to measure the elasticity. The smaller the value itself, the better the resilience performance of the material. Therefore, the reduction of the tensile permanent deformation of each group of materials after adding KH-172 means the improvement of the elastic properties of the material. After calculation, compared with the mechanical data without KH-172, the tensile strength is increased by about 25%, the elongation at the break is increased by about 15%, and the tensile permanent deformation is improved by about 20%. Combined with the characterization results of the size of the compatible layer in Figure 7, we believe that the improvement of the tensile and elastic properties of this modified TPV blend system by KH-172 is due to the increase in the size of the compatible layer, that is, KH-172 can increase the interaction between the two phases in the TPV system to a certain extent. From Figure 8 and Figure 9, in addition to the effect of KH-172, comparing the mechanical property data of each group with the different vulcanizing agent content, we found that when the vulcanizing agent content was 1 phr, the tensile strength was the largest, the tensile permanent deformation was the smallest, and the elongation at the break decreased gradually with the increase of the content of the vulcanizing agent. The decrease in elongation at the break was mainly related to the increase in the degree of the internal cross-linking of the polymer. As the cross-linked structure becomes denser, the degree of stretching of the polymer chain would decrease, and this phenomenon occurred. However, the tensile strength and elastic properties first improved with the increase of the vulcanizing agent content, and then the properties decreased greatly, both of which were mainly related to the degree of crosslinking and the size of the dispersed phase. When the amount of vulcanizing agent was increased from 0 phr to 1 phr, both the PSR and SBS were cross-linked to form a three-dimensional network structure. At the same time, it can be seen from Figure 5A–C that the size of the dispersed phase was significantly reduced, which made the material show good elasticity of PSR and good tensile properties of SBS-SEBS. However, as the amount of vulcanizing agent continued to increase, both the PSR and SBS demonstrated over-vulcanization. At the same time, it was difficult to make the shear force of the rotor disperse the dispersed phase well, resulting in a significant increase in the size of the dispersed phase. This can be seen from Figure 5D,E, where the overall tensile property and elasticity of the material declined significantly.

### 3.4. Analysis of the Experimental Results of Dynamic Mechanical Test and Low-Temperature Hardness

**Figure 10:**
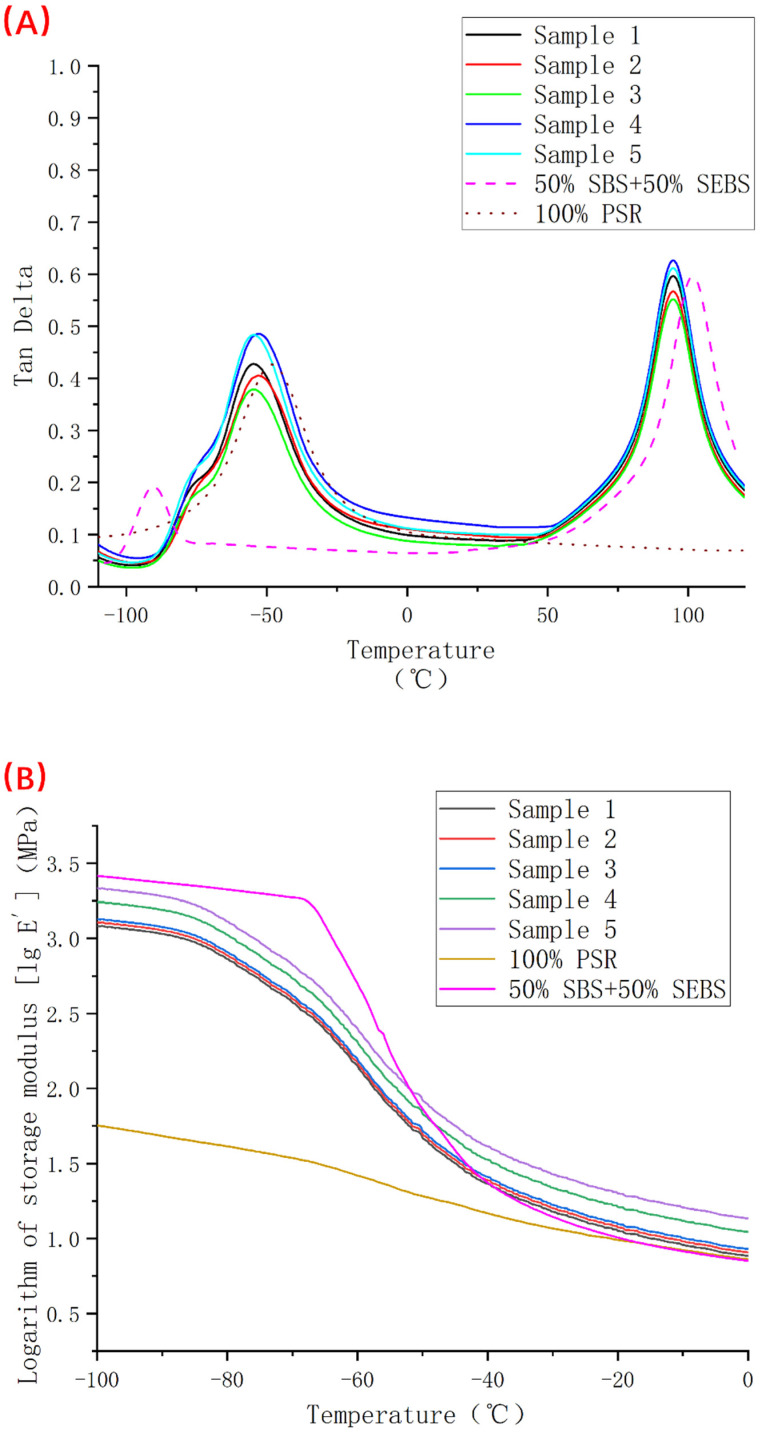
Dynamic mechanical test results of samples 1–5 (where (**A**) is the tangent value curve of loss angle from −110 °C to 120 °C and (**B**) is the logarithm of the storage modulus from −100 °C to 0 °C).

As shown in Figure 10A, a graph of the loss tangent value of samples 1–5, pure PSR and SEBS-SBS blends containing 50% of each. From the figure, we see that the blend of SEBS-SBS has two peaks, corresponding to the temperatures of −90 °C and 102 °C, respectively. These two peaks represent the polybutadiene segment and the polystyrene segment, respectively. The pure PSR peaks at −48 °C reflect the glass transition temperature of PSR. There are three peaks in the loss tangent curve corresponding to samples 1–5. Because both PSR and SBS exist in the sample, the peak of the glass transition temperature of PSR and the peak of the polybutadiene section of SBS appear. Although there is a certain degree of overlap, the peak temperatures of −75 °C, −55 °C, and 94 °C can also be distinguished. Compared with the pure components in the sample, the peaks of the polybutadiene segment of SBS are shifted by nearly 15 °C, the peaks of the polystyrene segment are shifted by nearly 8 °C, and the peaks of PSR are shifted by nearly 7 °C. Especially in the low-temperature region, the peaks of the two components are close, which indicates that the two components in the modified TPV system have a certain degree of compatibility.

Figure 10B shows the storage modulus curves of samples 1–5. To facilitate the observation of the changes in the magnitude of the storage modulus, we took the logarithm of the storage modulus value. The storage modulus is usually used to measure the elasticity of elastomers. People often regard the storage modulus as an index of the ability of materials to store elastic deformation energy. This concept is simply the ratio of stress to elastic deformation. According to the description of the definition, we find that under the same stress, the larger the storage modulus, the smaller the elastic deformation. If this phenomenon occurs at low temperatures, it means that the resilience of the rubber becomes poor. This can limit the use of the material at a certain temperature. From Figure 10B, we see that when the temperature is higher than −20 °C, the storage modulus of the 5 groups of samples is less than 10 MPa. However, the value of the storage modulus increased by an order of magnitude when the temperature dropped to −60 °C. The storage modulus increases by another order of magnitude when the temperature continues to drop to −80 °C. In addition, we compared the storage modulus curves of samples 1–5 with those of pure PSR and unvulcanized SBS/SEBS blends and found that the storage modulus of silicone rubber is less than 100 MPa in the entire temperature range. This indicates that PSR has good low-temperature resistance, that is, it can maintain good elasticity at low temperatures. The storage modulus of the unvulcanized SBS/SEBS blends increases sharply at around −40 °C, and the storage modulus exceeds 1000 MPa when the temperature reaches nearly −65 °C. Combined with the above analysis of the magnitude change of the storage modulus of samples 1–5, we also see that the addition of PSR can significantly improve the low-temperature performance of SBS/SEBS.

**Figure 11:**
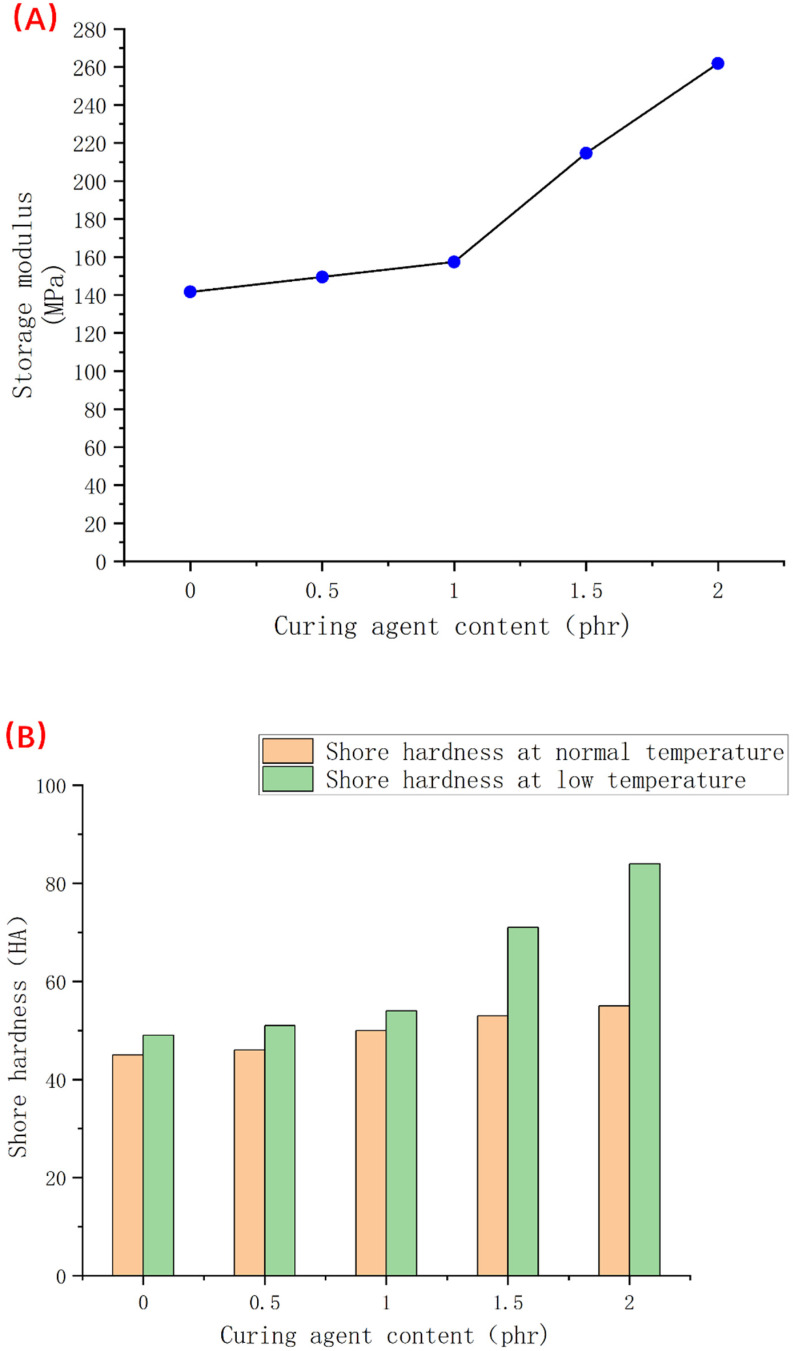
Low-temperature hardness change of samples 1–5 at −60 °C and broken line statistic diagram of the storage modulus at −60 °C ((**A**) is the broken line statistic diagram of the storage modulus at −60 °C and (**B**) is the column diagram of low-temperature hardness change at −60 °C).

We counted the storage modulus values of each group at −60 °C, as shown in Figure 11A, and also tested the hardness changes at room temperature and −60 °C, as shown in Figure 11B. From Figure 11B, we see that when the amount of the vulcanizing agent is more than 1 phr, the hardness of the material will increase sharply; in other words, the elasticity of the material will have significantly deteriorated. Combined with the data in Figure 11A, we found that the storage moduli of samples 4 and 5 with significantly increased hardness were both greater than 200 MPa. This means that in the temperature range where the storage modulus is greater than this value, the material will lose elasticity to a certain extent. At the same time, we also easily find from Figure 11A that with the addition of too much vulcanizing agent, the storage modulus of the material at low temperature will increase significantly, that is, the low-temperature performance will decrease. The degree of crosslinking is too deep, thus the importance of proper control of the amount of vulcanizing agent can be seen.

**Figure 12:**
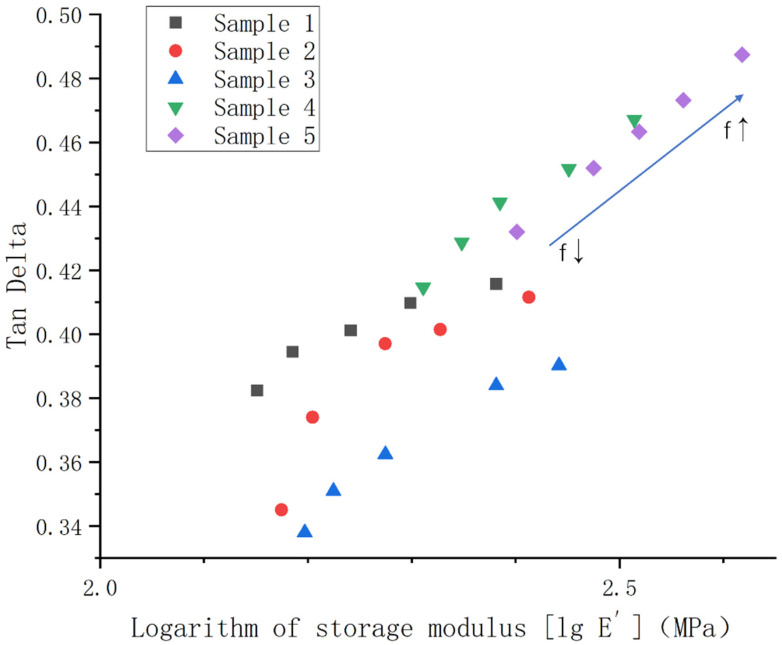
Relationship between the loss factor and storage modulus of samples 1–5 at −60 °C.

Furthermore, as shown in Figure 12, we plotted the loss factor versus the logarithm value of the storage modulus E’ at different frequencies (frequency range 1–20 Hz) at −60 °C. In this way, we observe the dependence of the response time and the temperature of the material under dynamic stress well [38,39]. From the figure, we see that there is a correlation between the loss factor and the storage modulus at different frequencies for each group of samples at −60 °C. Moreover, this correlation persists regardless of the vulcanization content, which is directly related to the degree of cross-linking. In addition, the storage modulus of each group of samples increases with the increase in frequency. According to the time-temperature equivalent principle, increasing the frequency has the same effect as lowering the temperature or reducing the force time. Therefore, the rigidity of the materials is increased, that is, the storage modulus is increased. It also provides a reference for the selection of the suitable application environments of materials.

### 3.5. Analysis of Melt Index Test Results

**Figure 13:**
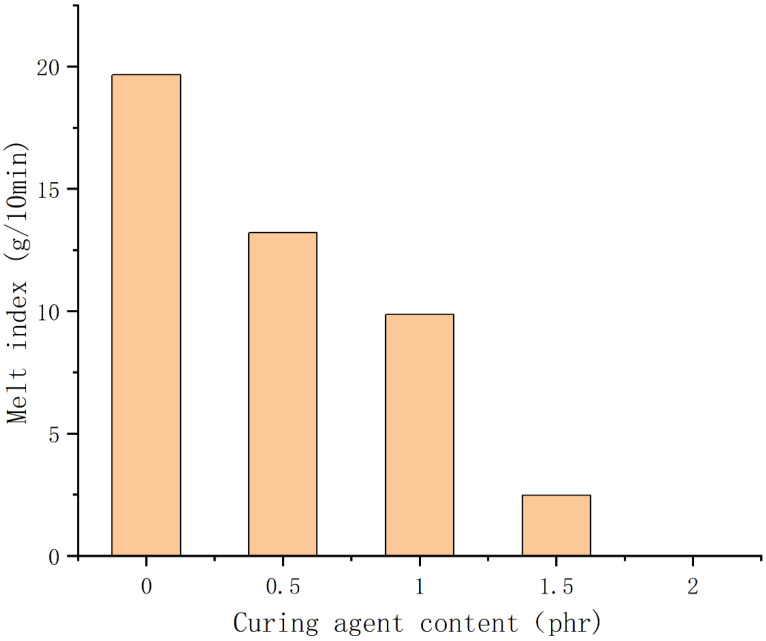
Histogram of the melt index of samples 1–5.

Figure 13 is a bar graph of the melt index of samples 1–5 tested under a load weight of 5 kg. The figure shows that the melt index can reach 19.66 g/10 min when no vulcanizing agent is added, which demonstrates that the modified TPV system has good fluidity during simple blending and is relatively easy to be extruded during processing. When the vulcanizing agent content increased from 0 phr to 1 phr, the melt index decreased by 32.81% and 25.36%, respectively. The main reason for this phenomenon is that with the increase of the vulcanizing agent content, the degree of cross-linking inside the two phases of the blend increases continuously, and the three-dimensional grid structure becomes more and more compact, which greatly reduces the fluidity of the blend. Then, when the vulcanizing agent content continued to increase to 2 phr, the melt index decreased by 74.85% and 100%, respectively. That is, when the vulcanizing agent content is 2 phr, the blend cannot be extruded from the melt flow rate tester. Combining Figure 5D,E, we see that the size of the PSR as a dispersed phase is very large, and the degree of crosslinking is too large, which makes it difficult for the PSR phase to be divided into smaller sizes by the shear force of the rotor, which also makes it difficult for the melt blend containing the PSR phase with the larger size to be extruded, or even impossible to be extruded. The melt index is an important indicator for determining processing technology. The data we tested here can provide a certain reference for the process selection of the products made from this blend system.

## 4. Conclusions

In summary, we successfully prepared a modified TPV using PSR/SEBS/SBS, which exhibited good mechanical properties and low-temperature properties. For the formulation of this material, we deeply studied the effect of the amount of vulcanizing agent on the material properties and microscopic phase distribution. It was found that when the dosage of the vulcanizing agent was 1 phr, the properties of the material were the best, the size of the dispersed phase was the smallest, and the dispersion of the dispersed phase was the best. In addition, through the analysis of the backscattered images and the test of the mechanical properties, we found that the silane coupling agent KH-172 can also significantly improve the tensile properties and elastic properties of this TPV system. Finally, we tested the thermal fluidity of the material and found that when the vulcanizing agent was used in excess of 1 phr, the fluidity of the melt was greatly reduced. In general, the new TPV we developed and prepared is expected to expand the application scenarios of silicone rubber products, and also provide a new idea for the development of similar products.

## Figures and Tables

**Table 1:** TPV dynamic vulcanization experimental formulations (unit: phr).

Entry	PSR	SEBS	SBS	250N	KH-172	Platinum Catalyst	Hydrogen-Containing Silicone Oil
1	50	25	25	10	1	0.5	0
2	50	25	25	10	1	0.5	0.5
3	50	25	25	10	1	0.5	1
4	50	25	25	10	1	0.5	1.5
5	50	25	25	10	1	0.5	2

**Table 2:** Characteristic peaks, corresponding groups, and vibration types of SBS.

Wavenumber (cm^−1^)	Designation of IR Spectra
541	Styrene block, deformations of the aromatic ring
700, 756	Out of plane deformations of the aromatic ring
720	-(CH_2_)- rocking vibration, -(CH_2_)_n_-, n, number of consecutive -CH_2_- groups are more than 4 in SEBS, the same as in polyethylene
738	Out of plane deformation of cis double bonds overlapped by peak 756 cm^−1^ from an aromatic ring
910	δ-_c-H_, =C-H, out of plane deformations of vinyl units
966	Out of plane deformation of trans-unit
1379	-CH(CH_2_CH_3_)CH_2_- unit, -CH_2_ for hydrogenated products SEBS
1453	Deformation of aromatic ring
1470	Asymmetric vibrations and deformations of groups -CH_2_, -CH_3_ in SEBS
1492, 1602	Stretching vibration of the double bond of the aromatic ring
1641	Stretching vibration of the double bond of vinyl units

## Data Availability

The data presented in this study are available on request from the corresponding author.
